# Identifying the insured and uninsured in rural America: an empirical discriminant analysis

**DOI:** 10.3934/publichealth.2021032

**Published:** 2021-05-17

**Authors:** Promise Tewogbola, Norah Aung

**Affiliations:** 1 School of Psychological and Behavioral Sciences, Southern Illinois University, Carbondale, Illinois, USA; 2 Department of Health Sciences and Social Work, Western Illinois University, Macomb, Illinois, USA

**Keywords:** uninsured, health insurance, insured, Affordable Care Act, Obamacare

## Abstract

**Purpose:**

This present study sought to investigate whether there were factors that could discriminate insured from uninsured rural Americans.

**Methods:**

Data for four groups were used: 34 uninsured, 102 government-insured (GP), 324 private- or employer-insured (PEP), and 96 both government- and private- or employer-insured (GPEP). A discriminant analysis was conducted on the four groups, using group membership as the dependent variable; age, education, income, attitude to insurance, emergency room visit, chronic disease prevalence were the independent variables.

**Findings:**

The analysis yielded three discriminant functions, however the only significant function was the one that discriminated the PEP-insured individuals from the other groups. About 48% of the cases were classified correctly with the significant discriminant function.

**Conclusion:**

The findings of this study can serve as a baseline for future research seeking to eradicate barriers to getting health insurance among the uninsured in rural America.

## 1. Introduction

Individuals resident in rural regions of the US find it challenging to access healthcare due to the fewer healthcare providers in rural regions, limited health insurance coverage provided by employers, long distance to healthcare facilities, among other reasons [1],[2]. The Affordable Care Act (ACA) was enacted to address some of these challenges and consequently make healthcare more accessible to individuals, irrespective of whether they were resident in urban or rural areas. This was achieved by making provisions to ensure that insurance companies were unable to use price discrimination tactics to discourage people with pre-existing medical conditions from purchasing insurance coverage. In addition, the qualification process for accessing government-funded insurance for individuals below the poverty line was made to be less restrictive. All health insurance plans were also expanded to provide additional health benefits, such as mental health services, as well as preventative care [3].

Despite the strides made by the ACA to improve access to healthcare, people continue to remain uninsured. For instance, Cha and Cohen [4] reported that 14.5% of adults in the US were uninsured for reasons ranging from not wanting coverage, to finding the signing up process too difficult. In addition, in the 2018 data obtained from a clinical data research network of community health centers (CHC), 23% of the 28 million patients were uninsured. Furthermore, in comparison with nonelderly individuals who are resident in urban areas, nonelderly individuals in rural America continue to be uninsured at a higher rate [5].

Although the consensus is that, generally speaking, rural Americans have higher uninsured rates than their urban counterparts do, few studies have investigated the specific factors that predict whether a rural resident will be insured or not. This present study attempts to fill that void in knowledge by answering two questions: First, whether there are variables that can reliably predict whether a rural resident is insured or not; and second, based on the discriminant functions created by those variables, what proportion of cases will be correctly identified. An understanding of the factors that distinguishes the insured from the uninsured in rural America can be useful in assisting policymakers and key decision makers in further reducing barriers to obtaining health insurance among the uninsured. This will be crucial to seeing the number of uninsured rural Americans drop, even as more Americans enjoy access to quality healthcare, regardless of their location.

## 2. Method

### 2.1. Study data

Secondary data was obtained from a predominantly white, rural county in a Midwestern state, where the local health department had conducted a health needs assessment survey. After dropping participants with missing responses, the total usable *N* was 556 (444 women, 110 men and 2 unspecified; 34 uninsured, 102 government-insured (GP), 324 private- or employer-insured (PEP), and 96 both government- and private- or employer-insured (GPEP)).

### 2.2. Measures

The needs assessment survey was designed and administered by the county health department. After providing consent, participants completed either the written or electronic form of the survey. The survey was conducted to fulfil an administrative requirement, rather than for the sole purpose of research. As a consequence, assessments of the reliability and validity of measures were unavailable.

**Age:** This describes the age of the participant. Choices ranged from *“Less than 18 years”* to *“65+”*. This was coded as “0” to “5”. Though data was collected as categorical, this variable was treated as continuous for analyses.

**Education:** This describes the highest level of education attained by the respondent. Choices ranged from *“High school without diploma”* to *“Graduate level degree”*, which were coded as “1” to “7”. This variable was also treated as continuous for analytical purposes.

**Income:** This describes the respondent's combined household income for the previous year. Choices ranged from *“Less than $20000”* to *“$100000 or more”* which was coded as “1” to “5”. This variable was also treated as continuous for analysis.

**Attitude towards insurance:** This assesses the respondent's perception of top insurance-related problems in the community. The score was computed from respondents' answers to the following questions: (1) Whether the respondent sees the ability to pay for care as one of the most pressing problems in the community; (2) Whether the respondent sees a lack of health insurance as one of the most pressing health problems in the community; (3) Whether the respondent sees a lack of health insurance as one of the most pressing health problems in the community. The scores ranged from *“0”* to *“3”* depending on the number of the aforementioned questions the respondent answered in the affirmative.

**Emergency room visit:** This asks how many times in the past 12 months the respondent or someone in the respondent's household had used a hospital emergency room. Choices available to respondents were *“None”, “1–2 times”, “3–5 times” and “6 or more times”* which was coded as *“0”, “1”, “3”* and *“5”* respectively. This variable was also treated as continuous for analysis.

**Chronic disease prevalence:** This asks about the number of chronic diseases such as hypertension, diabetes, and obesity, which the respondent currently has. Scores are weighted based on their prevalence rates in the US as reported by Buttorff, Ruder, and, Bauman [6].

**Insurance status:** This asks whether or not the respondent has insurance coverage, as well as the type of insurance coverage he/she has. The available categories were *“uninsured”, “government-provided (GP) insurance”, “private- or employer-provided (PEP) insurance”,* and *“both government- and private- or employer-provided (GPEP) insurance”*.

### 2.3. Statistical analysis

A direct discriminant analysis was performed using six variables as predictors of membership in 4 groups. Predictors were age, highest education level, income, attitude towards insurance, time of most recent visit to hospital emergency room, and chronic disease prevalence scores. Groups were uninsured, government-provided (GP) insurance, private- or employer-provided (PEP) insurance, and both government- and private- or employer-provided (GPEP) insurance. A discriminant analysis was used instead of a multivariate analysis of variance (MANOVA), because the research focused on predicting membership in naturally occurring groups, rather than those formed by random assignment. IBM SPSS statistical package (v. 25) was used for all analyses.

## 3. Results

Three more cases were excluded from the analyses because they were missing at least one discriminating variable. However, the characteristics of the dropped cases did not significantly differ from the cases eventually analyzed. Missing data appeared to be randomly scattered throughout groups and predictors. For the remaining 553 cases, evaluations of assumptions of multicollinearity were satisfactory. Z-scores for skewness for highest education level, income, time of most recent visit to hospital emergency room, and chronic disease prevalence scores were greater than 3.3 and were consequently transformed to approach normality. Additionally, statistically significant homogeneity of variance-covariance matrices (*p < 0.10*) was observed.

ANOVA results indicated that age (F(3,549) = 27.37, *p < 0.001*), highest education level (F(3,549) = 8.62, *p < 0.001*), income (F(3,549) = 26.72, *p < 0.001*), time of most recent visit to hospital emergency room (F(3,549) = 3.54, *p < 0.05*), and chronic disease prevalence scores (F(3,549) = 0.83, *p < 0.001*) all differed statistically between groups.

Three discriminant functions were calculated, with a combined Wilks' Lambda = 0.729, X2(18, N = 553) = 172.87, *p < 0.001*, accounting for 27.1% of the relationship between the predictors and groups. After the removal of the first and then the second function, the combined Wilks' Lambda increased to 0.978, χ^2^(10, N = 553) = 12.33, *p = 0.26*, and then 0.992, χ^2^(4, N = 553) = 4.59, *p = 0.33*. Canonical R = 0.504 for the first discriminant function, 0.119 for the second discriminant function, and 0.091 for the third discriminant function. Eigenvalue scores for each of the three discriminant functions were 0.341, 0.014 and 0.008 which respectively accounted for 93.8%, 3.9% and, 2.3% of the between-group variability.

The first discriminant function separated the PEP-insured from the other three groups, while the second discriminant function separated the uninsured from the other three groups. Since the Wilks' Lambda for the second and third discriminant functions were not significant, they are not be interpreted, although their functions at group centroids are shown in Table 1. The structure matrix of correlations between predictor and discriminant functions seem to suggest that the best predictors for distinguishing between the PEP-insured and the other groups (first discriminant function) are age (0.654), income (−0.648) and chronic disease prevalence scores (0.478). Because these variables are all related to events more likely to occur at a specific phase of life, this first dimension was labelled “chronophilic factors”. The PEP-insured were younger (mean = 3.85, SD = 1.03) than the uninsured (mean = 4.64, SD = 1.37), the GP-insured (mean = 4.90, SD = 1.51), and the GPEP-insured (mean = 4.74, SD = 1.41). Additionally, the PEP-insured were higher earners (mean = 3.37, SD = 1.61) than the uninsured (mean = 2.97, SD = 1.49), the GPEP-insured (mean = 2.43, SD = 1.64) and the GP-insured (mean = 1.95, SD = 1.12). Furthermore, the PEP-insured had lower chronic disease prevalence scores (mean = 6.55, SD = 10.48) than the uninsured (mean = 7.28, SD = 7.89), the GP-insured (mean = 13.73, SD = 13.95) and the GPEP-insured (mean = 13.25, SD = 13.31).

**Table 1.** Unstandardized canonical discriminant functions evaluated at group means.

Group	Function		
	1	2	3
Uninsured	0.136	0.461	−0.077
GP-insured	0.884	−0.076	−0.121
PEP-insured	−0.472	−0.027	−0.006
GPEP-insured	0.610	0.011	0.175

Thus, the four groups differ most notably on their age, income and chronic disease prevalence scores. Individuals who are PEP-insured are the highest earners, followed by the uninsured, the GPEP-insured and the GP-insured. Also, individuals who are PEP-insured tend to be younger than all other insurance groups and are less likely to be bothered by chronic diseases when compared with other groups.

As shown in Table 2, the classification analysis for the total usable sample of 553 respondents showed correct classification in 267 cases (48.3%). Although 58.4% of the respondents were PEP-insured, the classification scheme classified 35.1% of the respondents as PEP-insured (194/553 from the Cross-validation table). That said, PEP-insured respondents were still more likely to be correctly classified (60.1% correct classification) than the uninsured (48.5% correct classification), GP-insured (41.6% correct classification), and GPEP-insured (15.6% correct classification).

**Table 2.** Results of classification analysis.

Group	Predicted Group Membership				
	Number of Cases	Uninsured	GP-insured	PEP-insured	GPEP-insured
Uninsured	33	16 (48.5%)	6 (18.2%)	7 (21.2%)	4 (12.1%)
GP-insured	101	24 (23.8%)	42 (41.6%)	18 (17.8%)	17 (16.8%)
PEP-insured	323	64 (19.8%)	40 (12.4%)	194 (60.1%)	25 (7.7%)
GPEP-insured	96	20 (20.8)	32 (33.3%)	29 (30.2%)	15 (15.6%)

## 4. Discussion

The main objective of this study was to investigate whether there were discriminant functions that could distinguish insured rural Americans from their uninsured counterparts. The analyzed data did not reveal any significant discriminant functions delineating the insured from the uninsured. However, the discriminant function separating the PEP-insured from other groups can provide some insights that predict membership in this group, even though it accounted for about 27% of the variance in the groups.

The data analysis revealed that income, age, and prevalence of chronic diseases were the largest contributors to the discriminant function separating members in the PEP-insured group from the other groups. The analyzed data showed that the PEP-insured were the highest income earners. Since members in this group have higher levels of income in comparison to other groups, one can assume that they were not poor. On the other hand, members of the GP- or GPEP-insured groups were eligible for government-provided insurance because they were low-income earners, disabled, or elderly and are unable to command an income comparable to their counterparts in the PEP-insured group [7]. Studies have shown that poverty is one of the major contributors to a lack of insurance. For instance, Makowska [8] reported that 26% of the individuals below the US poverty line did not have health insurance. Wippold and colleagues [9] also provided evidence showing that people with low socioeconomic statuses struggle to “pay or co-pay...for services associated with health care”, which may further increase the likelihood of them delaying in seeking care as though they did not have insurance. Cha and Cohen [4] also reported that people's perception of their inability to afford coverage was a contributor to their status as uninsured.

Members in the PEP-insured group were also younger than their counterparts in other groups. This is plausible from the economic perspective of rural employers who may prefer to employ younger people because of their strength and potential for prolonged productivity. In exchange for their commitment to pursue employment in a rural region, younger people may be incentivized with health insurance coverage. This, however, is inconsistent with Makowska [8] who reported that people aged between 26 and 34 years were the most likely to be uninsured in comparison to other age groups.

Prevalence of chronic diseases was much lower in the PEP-insured group than in any other group. This may be because the accommodations of the PEP-insurance provides access to healthcare services that better helps this category of people to manage and ultimately cure their chronic diseases. Alternatively, members of the PEP-insured group may just be overall healthier than their counterparts in other groups, are potentially more productive economically, and are thus able to get insurance-providing employment. This is consistent with the suggestion of Fernandez-Lazaro and colleagues [10] that uninsured populations are more vulnerable to chronic diseases.

The current study has some limitations. As mentioned earlier, non-researchers designed the needs assessment survey for the purpose of fulfilling an administrative requirement. As a result, the validity and reliability of the survey tool could not be determined. Furthermore, data was self-reported by the respondents and analyses were conducted with the assumption that the survey questions were answered honestly and accurately. In addition, some of the variables of interest were collected as categorical, but analyzed as continuous. This, and the violation of some statistical assumptions, could affect the findings of the study, particularly the proportion of cases that the significant discriminant functions were able to predict accurately. Future research with data from more counties will be useful in providing more insight about the discriminant functions that separate insured, rural Americans from their uninsured counterparts. In the same vein, further studies can investigate discriminant functions that predict non-insurance when more rural counties are taken into consideration. Similar studies can also be conducted to find out the discriminant functions that can predict rural insurance coverage between genders, or amongst age groups.

## 5. Conclusions

Understanding the factors that discriminate insured rural Americans from their uninsured counterparts will prove to be vital to the creation of a future where everyone has access to healthcare. Although, the findings of this study did not show the factors that discriminated the uninsured from the insured, it did demonstrate that there were specific factors that distinguished the PEP-insured from others. By using this study as a baseline, and investigating further in future studies, policymakers and key stakeholders can design targeted interventions that can reliably increase insurance enrollment among uninsured, rural Americans.
